# Prevention of Iodism

**Published:** 1894-07

**Authors:** 


PREVENTION OF IODISM.—Dr. H. N. Spencer (*Int. Med. Mag.*, December, 1893) recommends the following mode, due to Professor Hardaway, of prescribing iodide of potassium; the tendency to coryza is counteracted by the nux vomica and ammonia citrate, while the tonics prevent depression:

℞ Iodide of potassium.................................... ℥ ss.

Citrate of iron,

Ammonium..........................................āā ʒ j.

Tincture of nux vomica.................................. ʒ ij.

Water.................................................. ℥ jss.

Compound tincture of cinchona to make up ℥ iv.

Dose, one teaspoonful in half a glass of water after meals. The quantity of iodide may be increased to any desired extent by adding the necessary amount of a saturated solution.

